# Overcoming Protein Orientation Mismatch Enables Efficient Nanoscale Light-Driven ATP Production

**DOI:** 10.1021/acssynbio.4c00058

**Published:** 2024-04-03

**Authors:** Andrea Marco Amati, Stefan Urs Moning, Sacha Javor, Sandra Schär, Sabina Deutschmann, Jean-Louis Reymond, Christoph von Ballmoos

**Affiliations:** Department of Chemistry, Biochemistry and Pharmaceutical Sciences, University of Bern, Freiestrasse 3, 3012 Bern, Switzerland

**Keywords:** energy conversion, synthetic biology, ATP synthesis, membrane protein orientation, liposomes, light-driven proton pumping

## Abstract

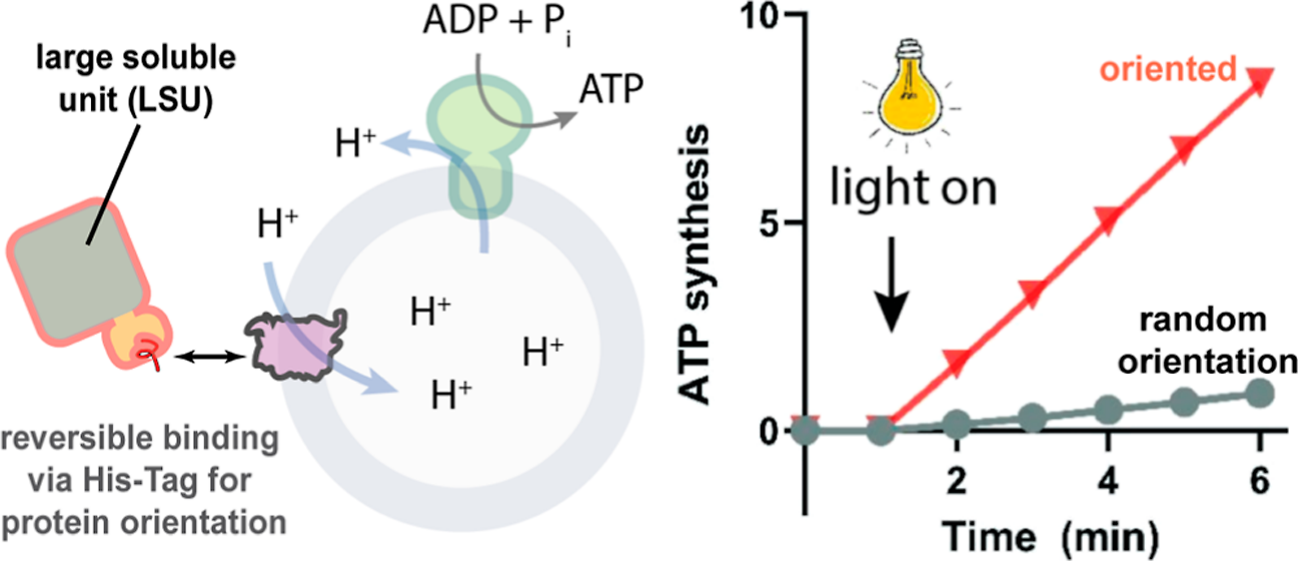

Adenosine triphosphate (ATP)-producing modules energized by light-driven proton pumps are powerful tools for the bottom-up assembly of artificial cell-like systems. However, the maximum efficiency of such modules is prohibited by the random orientation of the proton pumps during the reconstitution process into lipid-surrounded nanocontainers. Here, we overcome this limitation using a versatile approach to uniformly orient the light-driven proton pump proteorhodopsin (pR) in liposomes. pR is post-translationally either covalently or noncovalently coupled to a membrane-impermeable protein domain guiding orientation during insertion into preformed liposomes. In the second scenario, we developed a novel bifunctional linker, *tris*NTA-SpyTag, that allows for the reversible connection of any SpyCatcher-containing protein and a HisTag-carrying protein. The desired protein orientations are verified by monitoring vectorial proton pumping and membrane potential generation. In conjunction with ATP synthase, highly efficient ATP production is energized by the inwardly pumping population. In comparison to other light-driven ATP-producing modules, the uniform orientation allows for maximal rates at economical protein concentrations. The presented technology is highly customizable and not limited to light-driven proton pumps but applicable to many membrane proteins and offers a general approach to overcome orientation mismatch during membrane reconstitution, requiring little to no genetic modification of the protein of interest.

## Introduction

Adenosine triphosphate (ATP) is the universal energy currency of all cells, energizing a variety of processes such as muscle function, signaling processes, and nerve and brain function. Most of the cellular ATP is produced by the rotary F_1_F_O_ ATP synthase using an electrochemical gradient (proton motive force, *pmf*) to regenerate ATP from its hydrolysis products ADP and inorganic phosphate. Next to a variety of respiratory chains found in all kingdoms of life, decarboxylation reactions in anaerobic bacteria and light reactions in archaea, plants, and cyanobacteria are used as an energy source for the establishment of the required *pmf*.^1−3^ The simplest form of the latter category are rhodopsin-like light-driven proton pumps, small membrane proteins found in archaea and bacteria, using light to pump protons across a membrane.^4−6^

The coreconstitution of ATP synthase with the archaeal analogue bacteriorhodopsin (bR) was a seminal experiment by Racker and Stoeckenius to support Peter Michells’ hypothesis of chemiosmosis^7,8^ and has since been used as a minimal light-driven system to energize a variety of processes in bottom-up synthetic assemblies.^9,10^ This success is significantly owed to the fact that uncontrolled reconstitution of bR into preformed liposomes mainly yields a population acidifying the liposomal lumen by pumping protons to the inside,^7,11,12^ which is required for ATP synthesis on the outside of the liposomes, from where ATP can diffuse to its consumption sites. Drawbacks of bR are its poor heterologous expression^13^ and cumbersome molecular biology techniques in the native archaeal host *Halobacterium salinarium*.^14^ More recently, similar rhodopsin-like membrane proteins working as light-driven pumps for protons and sodium or chloride ions have also been found in bacteria and can be expressed as heterologous hosts in *Escherichia coli*. In addition to ion pumps, light-controlled channel rhodopsins were also found, which have found wide application and are the foundation of optogenetics.^15,16^

The bacterial proton-pumping analogue to bR is called proteorhodopsin (pR)^4,15^ and is well expressed in *E. coli* as a heterologous host. It can be easily genetically manipulated but inserts oppositely into liposomes, yielding a net outward pumping direction that is hence incompatible with liposomal ATP synthesis.^17,18^ Previous attempts to obtain inward-directed proton pumping include selective blocking of the outward-pumping population^19,20^ and N- and C-terminal fusion with either GFP or mCherry, respectively,^21^ guiding orientation with the fluorescent protein on the outside, in accordance to our earlier observation from experiments with the F_1_F_O_ ATP synthase.^22^

Here, we exploit this property with a modular approach using maltose binding protein (MBP) as a large soluble unit (LSU) to guide orientation of green-light-absorbing pR in the desired direction. Using the SpyTag/SpyCatcher technology,^23^ pR variants carrying a SpyTag either at the N- or C-terminus were expressed and purified to homogeneity. In parallel, MBP variants were fused to SpyCatcher, and the two proteins were coupled in vitro, purified, and reconstituted into preformed liposomes to maximize the effect of the LSU. Using kinetic proton pumping and membrane potential measurement, we show that the procedure guides directional insertion. If the inward pumping variant was coreconstituted with ATP synthase, pR-driven ATP synthesis was demonstrated for the first time. We obtained similar results by noncovalent coupling of the MBP moiety to the C-terminal His-tag of pR using a novel bifunctional linker consisting of a *tris*NTA moiety and the SpyTag peptide. This approach requires no modification of the target protein (except the HisTag), offers high flexibility toward the choice of the LSU, and allows for convenient removal of the LSU after reconstitution^24−26^ by the addition of histidine.

## Results and Discussion

### Purification of Proteins, Complex Assembly, and Reconstitution into Liposomes

**Figure 1:**
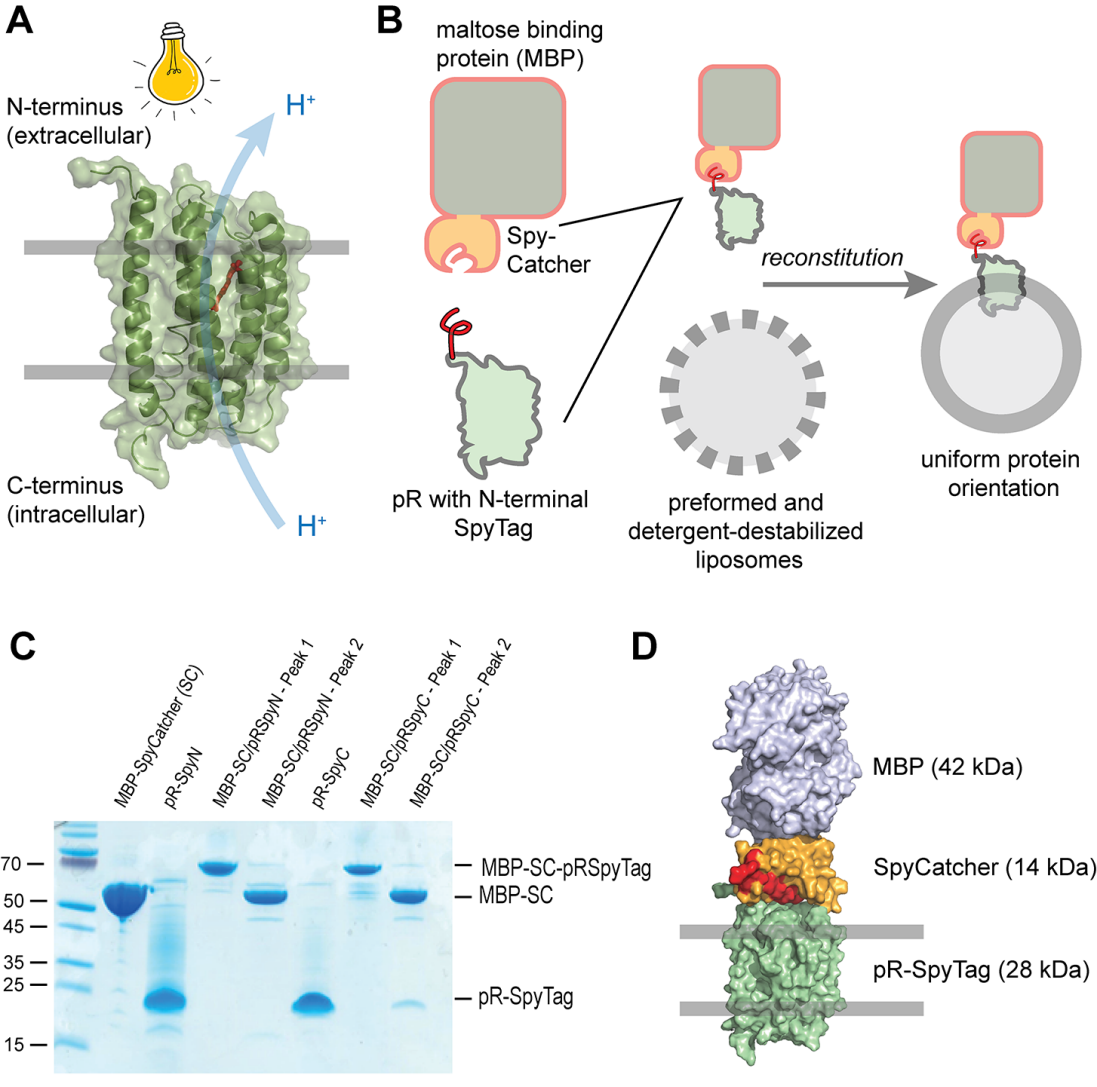
Overall experimental strategy and construct assembly. (A) Structure of the green-light absorbing pR (pdb: 2L6X). In its native orientation, the N-terminus is located on the extracellular side, and the proton pumping direction is from the C-terminal side to the N-terminal side, establishing an outside positive Δψ. (B) Cartoon representation of the protein orientation method. Spy-tagged pR and MBP-SpyCatcher are separately expressed, purified, and then mixed. The resulting construct is incubated with detergent-destabilized liposomes, and the detergent is removed using gel filtration. During the reconstitution process, the MBP moiety is expected to stay on the outside of the liposome, yielding a uniform pR orientation. (C) SDS-PAGE results of the different proteins expressed and purified. SpyCatcher-maltose binding protein (SC-MBP), pR with an N-terminal SpyTag (pR-SpyN), and pR with a C-terminal SpyTag (pR-SpyC). Also shown are also the elution peaks of the SEC after protein coupling (see Figure S2). (D) Surface representation of the final protein construct, composed of pR (green), SpyTag (red), SpyCatcher (orange), and MBP (purple) (pdb: 2L6X, 1N3W, and 4MLI). Assembly done in Pymol by eye.

Figure 1A shows a cartoon/surface representation of the pR structure (pdb: 2L6X) placed in a membrane bilayer^27^ and the cellular localization of its termini. Light-driven transport of protons from the inside to the outside of the cell establishes a *pmf* (inside negative) necessary for ATP synthesis and other cellular functions. Figure S1 shows the distribution of charged residues across the protein in accordance with the positive inside rule,^28^ explaining the preferential right-side out orientation during reconstitution guided by the interaction of the positive surface with the negative charges of the phospholipids.^18^ Based on earlier experiments with ATP synthase^22^ and protein fusion with pR,^21^ we speculated that an LSU attached to either side of the protein should guide the orientation as membrane penetration of a large hydrophilic unit is unlikely to happen during reconstitution with preformed liposomes (Figure 1B). Since genetic fusion of large subunits to membrane protein subunits often reduces protein yield and is not generally applicable, post-translational modification of the protein with a large soluble moiety was achieved using the SpyTag/SpyCatcher technology,^23,29^ allowing rapid and complete coupling between a 13-residue peptide (SpyTag) and the complementary 116-residue SpyCatcher domain by spontaneous isopeptide bond formation. Linkage works at a wide range of temperatures, pH values, and buffer compositions, and in the third and latest generation, the complete reaction within 30 min is reached with as little as 10 nM substrate proteins.^30^ The system has found wide application in connecting building blocks and surface display of proteins^31,32^ and in connection with virus-like particles (VLPs) and has been used to generate a multivalent platform for vaccine development.^33,34^

pR constructs containing the SpyTag 13-mer either N- or C-terminally of the protein sequence were expressed heterologously in *E. coli* and purified via Ni-NTA affinity chromatography. As an LSU, one or two copies of MBP from *E. coli* (∼45 kDa) were fused to SpyCatcher (∼14 kDa), followed by a thrombin-cleavable HisTag, yielding LSUs of ∼59 and ∼95 kDa, respectively.^34^ MBP was chosen for its high solubility, good expression yield, and overall negative surface charge (pI = 4.9) at physiological pH, expected to suppress attraction with phospholipid bilayers. pR was solubilized from membranes using 3% octyl glucoside (OG) and purified in buffer containing 1% OG to ensure a monomeric state of the enzyme as previously described.^21^ In the purified MBP-SpyCatcher constructs, HisTag was removed by thrombin cleavage, followed by reverse immobilized metal affinity chromatography (reverse IMAC) and size exclusion chromatography (SEC). Purified pR-SpyTag was mixed with a 1.2-fold excess of MBP-SpyCatcher for 2 h at room temperature, and the desired product was purified via SEC (Figure S2). The quality of the purified products and their coupling products after isopeptide bond formation was verified by using sodium dodecyl sulfate polyacrylamide gel electrophoresis (SDS-PAGE) (Figure 1C). An estimated assembly of the coupled product is depicted in Figure 1D. Reconstitution is based on protocols of Rigaud, Knol, and Poolman,^35,36^ in which preformed liposomes are destabilized at nonsolubilizing concentrations of detergent, forming a ternary complex with the added protein of interest before the excess detergent is removed using either gel filtration, polystyrene beads, or dialysis, listed here in the order of their relative removal time.^9^ After some optimization, we settled on a protocol combining a 30 min incubation of liposomes with the protein in the presence of 0.7% OG, followed by rapid detergent removal using short, prepacked SEC columns with a cutoff of 25 kDa, yielding protein reconstitution in <1 h, which is considerably faster than current protocols (>6 h).

### Light-Driven Proton Pumping, Membrane Potential Generation, and ATP Synthesis Measurements

**Figure 2:**
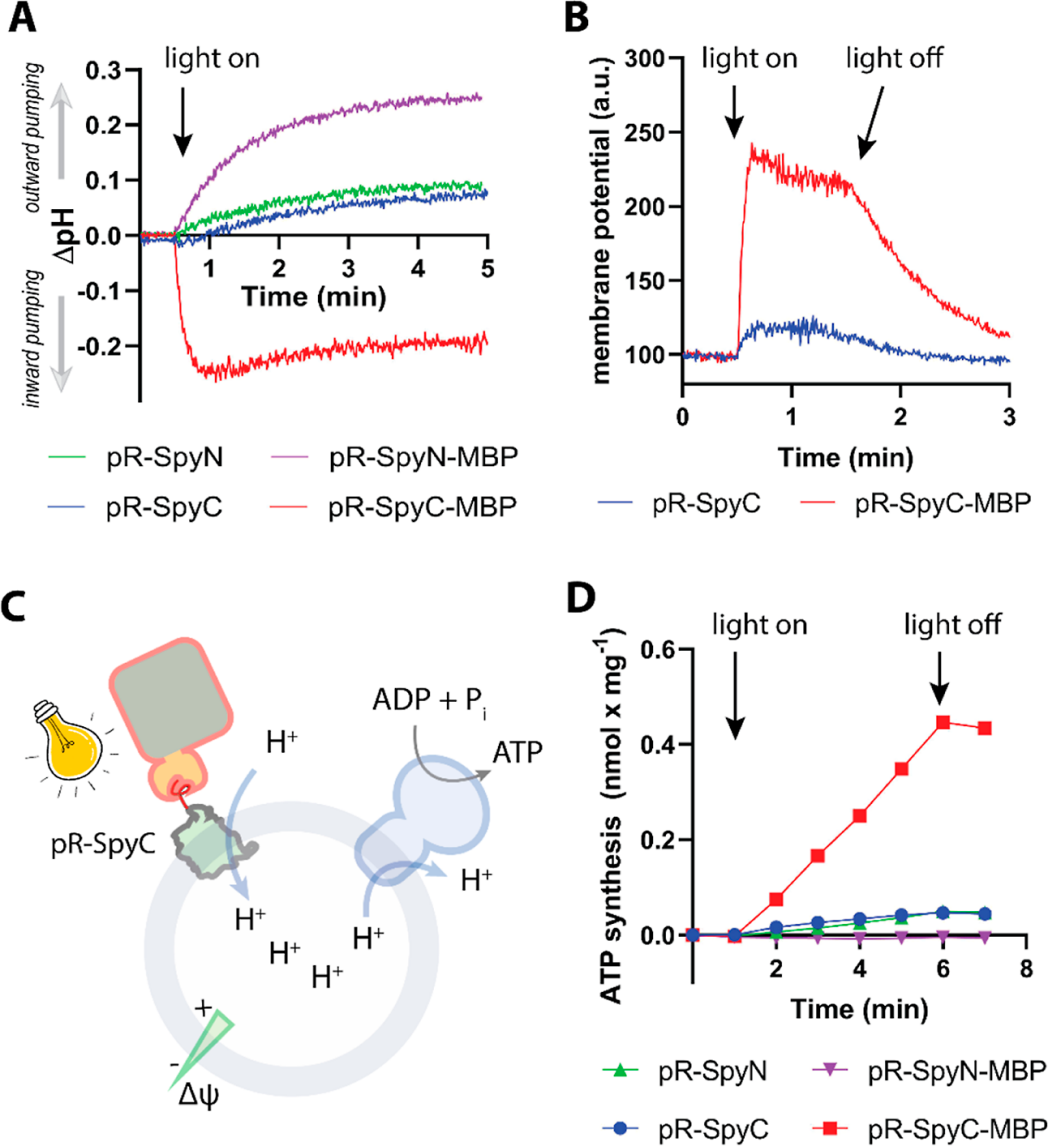
Proton pumping and ATP synthesis measurements. Shown are the data from representative experiments. The number of individual experiments (*n*) is indicated below. (A) Raw traces of light-driven proton pumping experiments using pyranine as a pH-sensitive dye. (*n* > 10). (B) Light-driven establishment of a membrane potential Δψ (positive inside) monitored using the fluorescent dye oxonol VI (*n* > 10). (C) Cartoon representation of the ATP synthesis experiments in liposomes containing different pR constructs and ATP synthase. Inward proton pumping of pR induced by illumination establishes a *pmf* used to drive ATP synthesis. (D) ATP synthesis of different pR constructs as described in C. At the indicated time points, illumination was stopped, and the ATP content was determined using a luciferin/luciferase-based assay. (*n* = 5).

To monitor pR activity, i.e., transmembrane proton pumping, the pH-sensitive fluorophore pyranine was entrapped in the liposomes during the reconstitution process, and excess dye was removed during subsequent gel filtration and ultracentrifugation as described.^22^ An aliquot of the pyranine-loaded proteoliposomes was transferred into a cuvette, and light driven proton pumping was initiated by illumination of the sample from the top with a custom-fitted LED lamp (520 ± 20 nm) (Figure S3). Thanks to the flashlamp design of the spectrometer, continuous illumination during fluorescence measurement is possible, and the ratiometric emission signal at 510 nm (excitation at 406 and 460 nm) was recorded. As depicted in Figure 2A, uncoupled N- and C-terminally Spy-tagged constructs yielded a slightly outward pumping signal in agreement with earlier reports.^18,21^ If the constructs were coupled to MBP as described above, the C-terminal construct changed its proton pumping direction to inward pumping, while the N-terminal pR-MBP construct showed an increased outward pumping activity, consistent with the guided orientation. In our experience, there was some variability in the amplitude of outward pumping activity of the noncoupled proteins in different reconstitutions, ranging from relatively mild (as depicted here) to much stronger outward pumping (see e.g. Figure 4B). A reason for this behavior might be a close to 50:50 orientation, which would yield no net pumping, probably due to our choice of phosphatidylcholine lipids minimizing electrostatic interactions with the protein during the reconstitution process.^18^ In contrast to earlier pR reports, a pH gradient was readily built up within less than a minute, indicating efficient proton pumping and yielding a rapid steady state of proton pumping and leakage. The size of the pH gradient depends on the pH of the experiment, being larger at pH 7.5 (ΔpH > −1) than at pH 6.75 (ΔpH ∼ −0.3) as shown in Figure 2A. Of special interest is the observed inward pumping profile of the construct with the C-terminal MBP attachment, which in conjunction with a coreconstituted ATP synthase^7^ should enable ATP synthesis. Consequently, the C-terminal MBP construct should establish a membrane potential Δψ that is positive and can be detected using the fluorescent dye oxonol VI.^37^ As depicted in Figure 2B, only in the presence of the MBP moiety is a substantial Δψ detected. The build-up is very rapid as only few charges are required to charge the membrane efficiently. Next, purified F_1_F_O_ ATP synthase from *E. coli*(22) was coreconstituted with the different pR constructs (Figure 2C), and ATP synthesis was detected by a luciferin/luciferase-based assay. As depicted in Figure 2D, only a small amount of ATP synthesis was observed in the two reconstitutions without MBP moieties (blue and green traces), which can be explained by a close to 50:50 orientation, as described above. In the C-terminal MBP-construct (red trace), ATP production was observed immediately, indicating an efficient energization of the vesicles with *pmf* enabling ATP synthesis to occur without delay. This observation is in accordance with the rapid buildup of a membrane potential, which is the major driving force of ATP synthesis in mitochondria and bacteria. No ATP synthesis was observed with the N-terminal MBP construct (magenta trace), in accordance with a purely outward pumping pR population. ATP synthesis measurements were quantified using a standardized amount of ATP added to the luciferase solution prior to the actual measurement. Based on this quantification, the ATP synthesis rate per ATP synthase per second can be estimated, reflecting the size of the imposed *pmf* by pR. This value, however, is strongly dependent on the number of coreconstituted proton pumps as they relate to the proton influx. Here, we use a molar pR to ATP synthase ratio of ∼20 (i.e., 20 pR and 1 ATP synthase per liposome), which is considerably lower than what has been used in earlier measurements (200:1).^8^ In a very recent report, Li et al. elegantly reported the coreconstitution of oriented bR and ATP synthase into microcapsules.^10^ Using a bR to ATP synthase ratio of ∼800, they calculated a total amount of 4500 nmol of ATP synthesized per hour per milligram of ATP synthase. With coreconstituted pR-SpyC-MBP and F_1_F_O_ ATP synthase, we find a similar amount of 3800 ± 500 nmol per hour per mg of ATP synthase despite using ∼40 times less pR per ATP synthase (20:1). While such calculations have several limitations and must not be overinterpreted, these estimates show that the present system is in efficiency comparable to or better than the bR/ATP synthase couple used in many applications. Further optimization of these numbers includes optimization of the reconstitution process (Figure S4) or increasing the pR number per vesicle, which can be adapted to the ATP requirement of the application. Varying the average number of pR between 12.5, 25, 50, and 100 per liposomes with a constant number of 3 ATP synthases on average, we find a direct relationship between the ATP synthesis rate and the number of pR molecules, which becomes nonlinear at higher pR numbers similar to what has been observed for bacteriorhodopsin^38^ (Figure S5A,B). As mentioned above, we also increased the size of the LSU to maximize the orientation effect and to suppress the undesired outward pumping direction of pR, which has a direct impact on the *pmf* and thus ATP synthesis. Comparative measurements of proton pumping and ATP synthesis of pR-SpyC linked to either MBP-SpyCatcher or 2xMBP-SpyCatcher are shown in Figure S6A,B. No substantial difference was observed in pH gradient formation and ATP synthesis, indicating that the size of a single MBP is sufficient to guide the orientation of pR.

### Noncovalent Attachment of an LSU Using *tris*NTA Technology

**Scheme 1:**
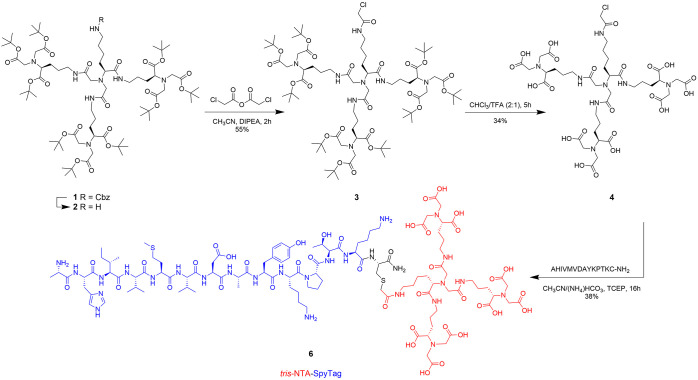
Synthesis of *tris*NTA-SpyTag 5 via Chloroacetyl–Sulfhydryl Coupling in Solution

The activity of pR is energized by a light-driven conformational change of the membrane internal retinal moiety that causes p*K*_a_ shifts of a few critical residues.^17^ It is thus not expected that the addition of an external moiety such as MBP will affect the overall protein activity. However, a covalent modification might be disadvantageous if membrane proteins are expected to interact with other molecules, as in structurally related G protein-coupled receptors (e.g., the epinephrine receptor), where binding of an external ligand induces the conformational change. While a potential solution would be the insertion of a protease cleavage site between the protein and SpyTag, this approach comes with a series of uncertainties. First, the insertion of the membrane protein into the liposome likely precludes efficient cleavage by a site-specific protease between two protein domains due to steric hindrance, as we have observed during our earlier efforts. Second, protease cleavage protocols typically require incubation times of several hours, during which protein inactivation is likely to happen. We reasoned that a temporary, noncovalent modification of a protein of interest with an LSU guiding the orientation during the reconstitution process that can be removed afterward, i.e., prior to functional experiments, would be a highly desirable development. To this end, we exploited the C-terminal HisTag in pR to orient the protein in a similar way to that with the covalently attached MBP-SpyCatcher moiety coupled to the C-terminus (pR-SpyC-MPB, see above). A HisTag typically ranges from 6 to 10 consecutive histidine residues and is the most frequently used protein affinity purification technology, in which the HisTag is bound to immobilized Ni^2+^-nitrilotriacetic acid (Ni^2+^-NTA) and released by imidazole or histidine. The affinity of the interaction is rather low (K_D_ ∼ 10 μM), which is ideal for protein purification but not suitable for experiments, in which a near 1:1 stoichiometry between HisTag and NTA is desired. A considerable improvement in affinity is obtained in molecules where three NTA moieties (*tris*NTA) are displayed on a circular or dendritic scaffold for which low nanomolar K_D_ values have been reported.^24−26^ Our strategy was to design a bifunctional linker molecule, harboring a high-affinity *tris*NTA moiety and a functional group which can be coupled to a large soluble protein moiety (see further discussion below). Here, for the *tris*NTA moiety, we chose a dendritic scaffold obtained by a relatively facile synthesis route leading to compound **1** (Scheme 1), as described by Huang et al.,^26^ in which the amino group and the NTA groups were protected as benzyloxycarbonyl and *tert*-butyl esters, respectively, allowing for selective deprotection of the amine by the Pd/C catalyst. The total deprotection is obtained by trifluoroacetic acid (TFA) and triisopropylsilane (TIPS). Motivated by our success with the SpyTag technology, we aimed to couple the *tris*NTA linker to a synthesized SpyTag peptide. As a coupling strategy, the primary amine **2** was activated with chloroacetic anhydride, enabling a facile reaction with the sulfhydryl group of a cysteine residue added C-terminally to the SpyTag peptide (Scheme 1).

**Figure 3:**
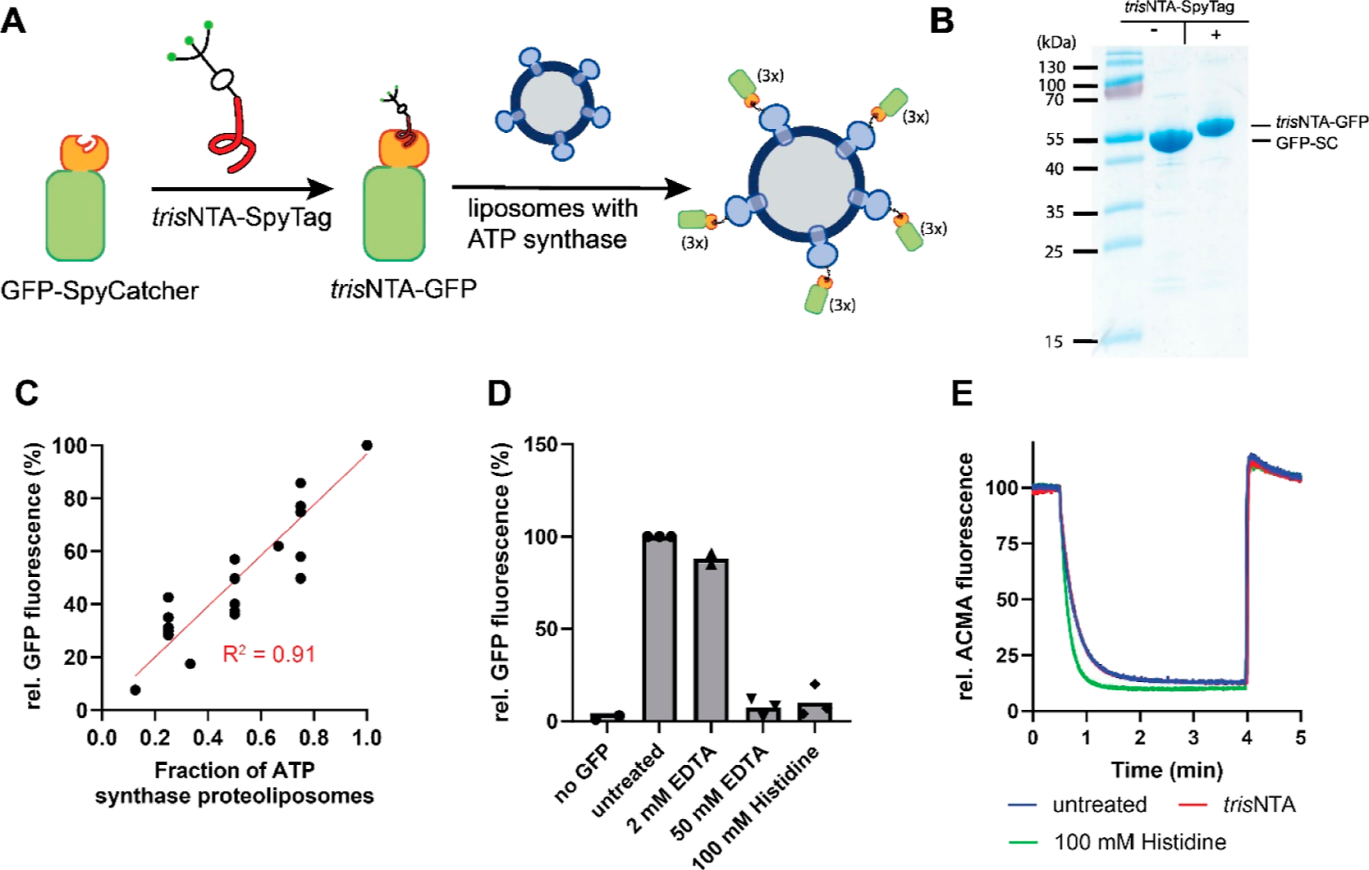
Reversible binding of *tris*NTA-GFP to ATP synthase reconstituted into liposomes. (A) Cartoon of the coupling strategy using noncovalent attachment of GFP to ATP synthase reconstituted in liposomes via HisTag–*tris*NTA interaction (see the text for details). (B) SDS-PAGE analysis of the coupling reaction of purified GFP-SpyCatcher with the synthesized peptide *tris*NTA-SpyTag (2.5 kDa). (C) Stoichiometric binding of *tris*NTA to varying amounts of proteoliposomes containing ATP synthases mixed with empty liposomes, keeping the total amount of lipids constant. After incubation for 15 min with a constant amount of *tris*NTA-GFP, proteoliposomes were collected by ultracentrifugation and resuspended, and GFP fluorescence was measured. (*n* = 3) (D) Liposomes were prepared as in (C) and either treated with the indicated amount of the elution agent or left untreated. Proteoliposomes were collected by ultracentrifugation and resuspended, and GFP fluorescence of the resuspended pellet was measured (*n* = 3). (E) ATP-driven proton pumping into liposomes was monitored using ACMA fluorescence. Shown are the traces of the untreated sample (before incubation with *tris*NTA-GFP), after incubation (in the presence of *tris*NTA-GFP), or after treatment with 100 mM histidine (Figure S7). After 30 s, the reaction was started with the addition of 2.5 mM ATP and stopped after 4 min by the addition of 60 mM NH_4_Cl (*n* = 2).

Following deprotection of intermediate **3** with TFA, the highly water-soluble compound **4** was purified by reverse phase high-performance liquid chromatography (RP-HPLC). After coupling with the SpyTag-Cys peptide in aqueous solution, the *tris*NTA-SpyTag conjugate, the final product **5** (*tris*NTA-SpyTag), was purified via RP-HPLC and used in subsequent experiments. In the first application, we wanted to verify that the synthesized peptide is active, reacts with a SpyCatcher-containing protein, and recognizes a HisTag on a second protein embedded in liposomes in a stoichiometric manner (Figure 3A). To this end, the purified *tris*NTA-SpyTag peptide (1 mM), charged with Ni^2+^, was incubated with 50 μM purified green fluorescent protein fused C-terminally to a SpyCatcher (GFP-SC), yielding *tris*NTA-GFP (Figure 3B). As a model protein, we reconstituted ATP synthase from *E. coli* equipped with a HisTag in the F_1_ part into proteoliposomes allowing for facile activity measurements at any experimental stage. We mixed proteoliposomes containing His-tagged ATP synthase with empty liposomes in different ratios, yielding a constant number of liposomes with varying amounts of ATP synthase per sample. The samples were incubated with a fixed amount of *tris*NTA-GFP, briefly incubated, and subjected to ultracentrifugation to collect liposomes, leaving unbound *tris*NTA-GFP in the supernatant. Subsequent analysis of the GFP fluorescence in the pellet fraction yielded values that reflected the relative ATP synthase content per sample (Figure 3C). Next, we tested different conditions to release *tris*NTA-GFP from reconstituted ATP synthase. To this end, proteoliposomes containing ATP synthase and *tris*NTA-GFP were mixed with either 2 mM EDTA, 50 mM EDTA, or 100 mM histidine and submitted to ultracentrifugation, and the fluorescence of the pellet was analyzed as above. Near complete release was observed in the presence of 50 mM EDTA or 100 mM histidine but not with 2 mM EDTA (Figure 3D). To test the compatibility of the *tris*NTA-GFP attachment and its subsequent release with enzyme functionality, ATP-driven proton pumping was measured before and after LSU release by 100 mM histidine. ATP-driven acidification of the liposomal lumen was monitored using the pH-sensitive fluorescent dye ACMA and showed that neither coupling with *tris*NTA-GFP nor its release affected protein function (Figure 3E).

### Orientation of pR Using *tris*NTA-MBP

**Figure 4:**
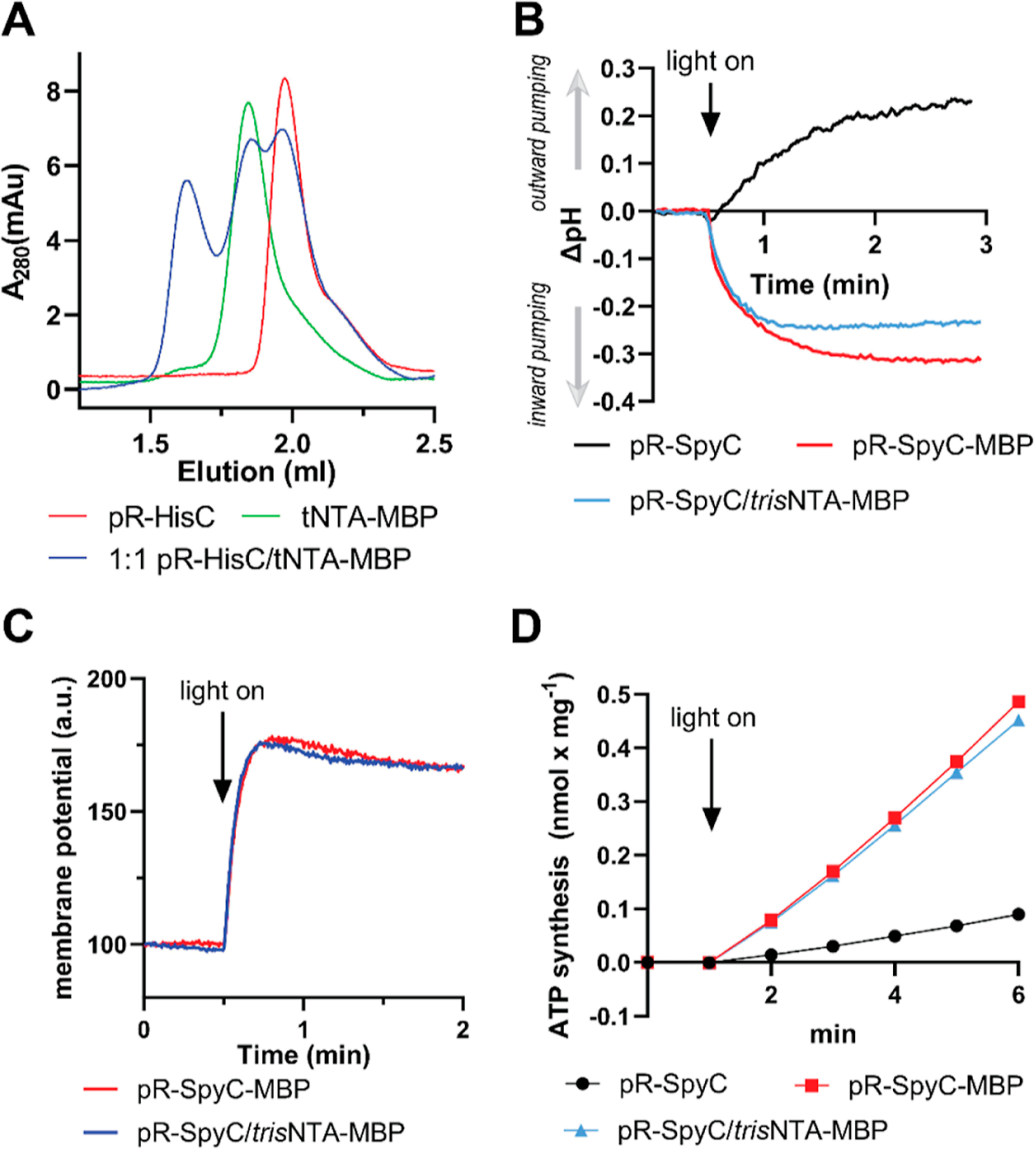
(A) Size exclusion chromatograms of the two substrates pR-HisC (red) and *tris*NTA-MBP (green) and a 1:1 mixture of both (blue) (*n* = 3). (B) Light-driven proton pumping monitored using pyranine entrapped in proteoliposomes containing different pR constructs (*n* = 3). (C) Comparison of light-driven membrane potential generation of covalently (red) and noncovalently (blue) coupled MBP to pR (*n* = 2). (D) Comparison of light-driven-ATP synthesis in proteoliposomes containing ATP synthase and different pR constructs (see the graph for legend) (*n* = 3).

Finally, we set out to apply the *tris*NTA-SpyTag technology to orient pR using its C-terminal HisTag, yielding the desired inward-pumping direction (Figure S8). As described above for GFP-SpyCatcher, the *tris*NTA-SpyTag peptide was coupled with the purified MBP-SpyCatcher protein to yield *tris*NTA-MBP and purified via SEC. Next, *tris*NTA-MBP was mixed with pR-HisC, a pR variant (without SpyTag) that contains a C-terminal HisTag in a 1:1 ratio, and the mixture was subjected to analytic SEC. As depicted in Figure 4A, the noncovalently coupled product eluted at ∼1.6 mL retention volume, while the two substrates eluted later (∼1.9 and ∼2.1 mL). The areas under the three peaks are similar, which is in accordance with earlier reports of similar experiments between *tris*NTA and HisTag peptides.^25^ For the remainder of the experiments, we replaced pR-HisC with pR-SpyC that also contains a C-terminal HisTag for better comparison with the results obtained with the covalent pR-SpyC-MBP construct discussed above. Based on the binding of a fluorescently labeled *tris*NTA-MBP to proteoliposomes containing pR-SpyC, a 5-fold excess of *tris*NTA-MBP was chosen for orientation experiments (Figure S9A). To evaluate the efficiency of *tris*NTA-MBP of guiding the orientation during the reconstitution process, uncoupled pR-SpyC, covalently coupled pR-SpyC-MBP, and pR-SpyC/*tris*NTA-MBP were reconstituted into proteoliposomes and collected by ultracentrifugation, and proton pumping was followed using pyranine. As depicted in Figure 4B, the rates and the extent of ΔpH generation were very similar for the covalent and noncovalent MBP constructs, while the uncoupled pR showed a very strong outward pumping in this experiment. The measurements were repeated using a varying excess of *tris*NTA-MBP over pR-SpyC, confirming that a 5-fold excess is sufficient for maximal activity (Figure S9B). Next, pyranine was omitted, and instead, Δψ generation was measured using oxonol VI, and the data in Figure 4C confirm that *tris*NTA-MBP can orient pR as efficiently as covalently coupled MBP. Finally, we coreconstituted the three different pR constructs with ATP synthase into liposomes and monitored light-driven ATP synthesis using the luciferin/luciferase-based assay (Figure 4D). In accordance with the other measurements, no significant difference between the covalently and noncovalently coupled MBP construct was observed.

A potential complication in this experiment might be that the ATP synthase also carries a HisTag, which competes with the pR-SpyC for *tris*NTA-MBP during the reconstitution process. However, the 5-fold excess of *tris*NTA-MBP over pR-SpyC used and the lower number of ATP synthases per liposome (1 ATP synthase compared to 20 pR) make this effect unlikely significant.

During the experiments above, *tris*NTA-MBP was not actively removed, and the observed rates indicate that the interaction does not interfere with the enzyme activity. Experiments with fluorescently labeled *tris*NTA-MBP showed that >70% of the LSU was already lost in the presence of 20 mM histidine (Figure S10A) and that no effect on activity on was observed up to 100 mM histidine (Figure S10B), where essentially all LSUs were removed.

## Conclusions

One of the cornerstones in bottom-up synthetic biology is the continuous supply of experimental systems with ATP, the universal energy currency of the cell. Not only can the rate of ATP production by light-driven proton pumps and ATP synthase be conveniently modulated by the light intensity, but the direct coupling between the two enzymes via proton transfer also omits the requirement of substrate regeneration or product removal, allowing maximal lifetimes. Here, we addressed the limitation of uncontrolled orientation of proton-pumping pR during reconstitution in liposomes with a general strategy using either a covalent or noncovalent modification with a large soluble protein moiety guiding protein orientation in the desired direction. Using this approach, we report for the first time the use of a light-driven pump different from bacteriorhodopsin for liposomal ATP synthesis. We find rates similar to those reported for bacteriorhodopsin, despite a ∼40-times lower protein concentration, expanding the toolbox for synthetic biology applications.

On a more general level, orientation of membrane proteins has been a longstanding challenge in membrane biochemistry, which previously had not been successfully tackled with a general method.^9^ The previous studies from us and others have shown that large extra-membranous soluble domains guide orientation, if detergent-stabilized preformed liposomes are used as the starting material.^9,22,39,40^ Our presented approach to add such a soluble unit post-translationally via SpyTag circumvents the requirement to design and express membrane proteins with large fusion domains, which might affect the expression yield or functionality. Here, we have used MBP and 2xMBP fused to SpyCatcher as units with molecular weights of ∼50 and 100 kDa with an overall negative surface charge, but proteins of any size or properties can be used as long as they contain a SpyCatcher domain. Importantly, the protein of interest requires only a short SpyTag and can be tested with all different coupling proteins, enabling efficient screening for successful orientation. If both N-terminus and C-terminus happen to be on the same side of the membrane, SpyTag can also be inserted within the protein, e.g., in a loop region,^41^ allowing us to attach the LSU at the desired side of the protein. Not only is the described method a solution for any other rhodopsins transporting alternative substrates such as sodium or chloride ions used in optogenetic research,^16^ but it is also expected to work for many other membrane proteins to be reconstituted, e.g., secondary substrate transporters or G protein-coupled receptors. For cases where the HisTag is on the desired side of the protein, we present the synthesis of a novel bifunctional linker *tris*NTA-SpyTag that connects two proteins with high specificity and affinity and allows for the convenient attachment or removal of an LSU without the requirement of genetic modification of the protein of interest. The interaction between of the dendritic *tris*NTA scaffold used here (with a reported *K*_D_ ∼ 10 nM with a HisTag peptide^26^) and the HisTag on pR is strong enough to survive a gel filtration column if mixed in a 1:1 ratio, which is not observed if a *mono*NTA linker is used.^24^ Because complex formation was not complete, we suggest using a 5-fold excess of the trisNTA moiety over the His-Tag protein. Complex formation might be improved using a more efficient trisNTA scaffold^25^ or by extending the His_6_-Tag on the pR construct. In combination with the attached SpyTag peptide, the bifunctional linker is a versatile tool allowing the coupling of any SpyCatcher-containing protein to the HisTag of a second protein. In our experience, the linker is stable for at least a year, if stored at −20 °C, without visible loss of integrity, as judged by mass spectrometry. We envision that the linker will find application not only for membrane protein orientation but also for a convenient colocalization of proteins in other scenarios, e.g., fluorescence microscopy or tethered proteins for improved catalytic rates in artificial biocatalytic pathways.^42^

## Supporting Information Available

Materials used; experimental procedures describing protein purification, construct assembly, and spectroscopic measurements; the detailed chemical synthesis route of trisNTA-SpyTag including NMR and mass spectrometry data; and supplementary results including additional experimental data comparing differently sized LSUs, binding and elution data of trisNTA-SpyTag to pR, as well as optimization of light-driven ATP synthesis using different reconstitution times and pR/ATP synthase ratios (PDF)
