# Supplementary material for: Overcoming Protein Orientation Mismatch Enables Efficient Nanoscale Light-Driven ATP Production
Source: ACS Synth Biol. 2024 Apr 3;13(4):1355–64. doi: 10.1021/acssynbio.4c00058 (PMC11036485; doi:10.1021/acssynbio.4c00058)
Supplement: Supplementary file 1 — sb4c00058_si_001.pdf [file sb4c00058_si_001.pdf]

Supporting Information

Overcoming protein orientation mismatch enables efficient nanoscale light-driven ATP production

Andrea Marco Amati[§,1], Stefan Urs Moning[§,1], Sacha Javor, Sandra Schär, Sabina Deutschmann[1], Jean-Louis Reymond and Christoph von Ballmoos*

Department of Chemistry, Biochemistry and Pharmaceutical Sciences

University of Bern, Freiestrasse 3, 3012 Bern, Switzerland

[1]Graduate School for Cellular and Biomedical Sciences, University of Bern, Bern, Switzerland

[§]These authors contributed equally to this work.

*To whom correspondence should be addressed: christoph.vonballmoos@unibe.ch

Table of contents

Supporting Information 1

Experimental Procedures 3

Materials & Plasmids 3

Amino acid sequence of proteorhodopsin constructs: 3

pR-SpyN 3

pR-SpyC 3

Expression and purification of pR-SpyTag constructs 4

Expression and purification of MBP-SpyCatcher and 2xMBP-SpyCatcher 4

Expression and purification of F_1F_0 ATP synthase 5

Expression and purification of GFP-ELP-SpyCatcher 6

Covalent coupling of SpyTag carrying proteorhodopsin variants and MBP-SpyCatcher variants 7

Synthesis of trisNTA-SpyTag 7

Synthesis of tris-NTA 7

Peptide Synthesis 7

Synthesis of tris-NTA-t-Bu-ClAc 3 8

Synthesis of tris-NTA-ClAc 4 10

Synthesis of tris-NTA-SpyTag 5 11

Non-covalent coupling of proteorhodopsin and LSU with bifunctional *tris*NTA-SpyTag system 12

Coupling of *tris*NTA-SpyTag with SpyCatcher containing LSU 12

Labelling of *tris*NTA-MBP with DY647P1 NHS-ester 12

Reversible coupling with *tris*NTA and HisTag 13

Proton Uptake Measurements with ACMA 13

Reconstitution of membrane proteins into preformed liposomes 13

Light-driven proton pumping measurements 14

Membrane potential measurements 14

Light driven ATP production 14

Results and Discussion 15

Surface charge of proteorhodopsin influences the orientation into liposomes 15

References 20

Experimental Procedures

Materials & Plasmids

If not otherwise stated, chemicals were obtained from Sigma. Plasmids containing proteorhodopsin were synthesized from Gene Universal (Newark, US). The plasmid carrying the sequence of two MBP copies and a C-terminal SpyCatcher (pET21 MPBx-SpyCatcher) from the Howarth lab [1] was ordered from Addgene (#72327). The plasmid containing only one MBP copy was derived from the previous plasmid by restriction digestion with Eco71I, purification of the long fragment and subsequent ligation. As the restriction site is located in the ORF of the MBP protein, this strategy leads to removal of one MBP unit. The correct sequence was confirmed using sequencing (Microsynth, Balgach, Switzerland).

Amino acid sequence of proteorhodopsin constructs:

pR-SpyN

MKYLLPTAAAGLLLLAAQPAMAMAHIVMVDAYKPTKSASDLDASDYTGVSFWLVTAALLASTVFFFVERDRVSAKW
KTSLTVSGLVTGIAFWHYMYMRGVWIETGDSPTVFRYIDWLLTVPLLICEFYLILAAATNVAGSLFKKLLVGSLVMLVF
GYMGEAGIMAAWPAFIIGCLAWVYMIYELWAGEGKSACNTASPAVQSAYNTMMYIIIFGWAIYPVGYFTGYLMGD
GGSALNLNLIYNLADFVNKILFGLIIWNVAVKESSNALEIKRASQPELAPEDPEDVEHHHHHH*

pelB Signal sequence SpyTag proteorhodopsin HSV-Tag His-Tag

pR-SpyC

MKYLLPTAAAGLLLLAAQPAMAMGSASDLDASDYTGVSFWLVTAALLASTVFFFVERDRVSAKWKTSLTVSGLVTGI
AFWHYMYMRGVWIETGDSPTVFRYIDWLLTVPLLICEFYLILAAATNVAGSLFKKLLVGSLVMLVFGYMGEAGIMAA
WPAFIIGCLAWVYMIYELWAGEGKSACNTASPAVQSAYNTMMYIIIFGWAIYPVGYFTGYLMGDGGSALNLNLIYNL
ADFVNKILFGLIIWNVAVKESSNALEIKRAHIVMVDAYKPTKASQPELAPEDPEDVEHHHHHH*

pelB Signal sequence SpyTag proteorhodopsin HSV-Tag His-Tag

Expression and purification of pR-SpyTag constructs

All pR variants were expressed in *E. coli* C43 cells. Expression cultures were grown in LB medium at +37 °C to an OD_{600} of approximately 0.9 in a LEX-48 bioreactor (Epiphyte Three, Toronto, Canada). Overnight protein expression at +30 °C was induced by the addition of 2 mM IPTG and 10 mg/L all-trans-retinal (Santa Cruz Biotechnology, Dallas, US). Cells were harvested by centrifugation and washed once with 50 mM MOPS pH 7, 300 mM NaCl prior to storage at –80 °C.

To prepare the membrane fraction, cells were disrupted by a high-pressure homogenizer HPL6 (MAXIMATOR AG, Düdingen, Switzerland) in the presence of DNase I, protease inhibitors PMSF, Pefabloc, and lysozyme. Cell debris was separated by centrifugation (12’000 g, 20 minutes, +4 °C). Membranes were collected by ultracentrifugation (235’000 g, 90 minutes, +4 °C) and resuspended in 50 mM MOPS pH 7, 300 mM NaCl, 5 mM imidazole.

The membranes were solubilized by addition of 3 % (w/v) *n*-Octyl β-D-glucopyranoside (OG, Glycon, Luckenwalde, Germany) at +4 °C overnight with mild stirring and centrifuged (257’000 g, 30 minutes, +4 °C) to separate insolubilized material. The supernatant was loaded on Ni-NTA agarose beads (PureCube, Monheim, Germany) preequilibrated with binding buffer (50 mM MOPS pH 7, 300 mM NaCl, 5 mM imidazole, 1 % OG). The column was washed with 10 CV binding buffer, 2 CV binding buffer containing 50 mM imidazole and finally eluted with the same buffer containing 250 mM imidazole. Colored fractions were concentrated (Amicon Ultra, 10 kDa cut-off, Merck, Darmstadt, Germany) and subjected to gel filtration (Superdex 200 Increase 10/300 GL, Cytiva, Marlborough, US) in 20 mM MOPS-BTP pH 7.25, 150 mM NaCl, 10 % glycerol, 1 % OG, 0.5 mM TCEP. Peak fractions were pooled and concentrated (Amicon Ultra, 10 kDa cut-off, Merck, Darmstadt, Germany).

Expression and purification of MBP-SpyCatcher and 2xMBP-SpyCatcher

Plasmids containing MBP-SpyCatcher and 2xMBP-SpyCatcher were transformed into competent *E. coli* BL21 pLysS cells and grown in LB media at +37 °C to an OD_{600} of 1.2 using a LEX-48 bioreactor

(Epiphyte Three, Toronto, Canada). Protein expression was started by the addition of 2 mM IPTG (Santa Cruz Biotechnology, Dallas, US) and incubation was continued for 3 more hours at +37 °C. The cells were harvested by centrifugation, washed with PBS, flash frozen in LN_2 and kept at –80 °C.

Cells were disrupted using a high-pressure homogenizer HPL6 (MAXIMATOR AG, Düdingen, Switzerland) in the presence of DNase I, protease inhibitors PMSF, Pefabloc, and lysozyme. Cell debris was removed by centrifugation (12’000 g, 20 min, +4 °C). The supernatant was supplied with 5 mM imidazole and loaded on Ni-NTA agarose beads (PureCube, Monheim, Germany), preequilibrated with PBS containing 5 mM imidazole. After 3 washing steps (5 CV PBS with 5 mM imidazole, 7 CV with 20 mM imidazole and 5 CV containing 50 mM imidazole) the column was eluted with PBS containing 250 mM imidazole. The HisTag was cut off overnight with 4 units of thrombin (Lee BioSolutions, Maryland Heights, US) per mg of protein at +4 °C. Thrombin was inhibited by 0.2 mM PMSF and removed with a 1 mL HiTrap Heparin column (Cytiva, Marlborough, US). The cut-off HisTag was removed with a 5 mL HisTrap FF column (Cytiva, Marlborough, US) equilibrated with PBS, 20 mM imidazole. The flowthrough was concentrated and subjected to a final size exclusion chromatography in PBS (Superdex 200 increase 10/300 GL, Cytiva, Marlborough, US). Peak fractions were pooled and concentrated (Amicon Ultra, 50 kDa cut-off, Merck, Darmstadt, Germany).

Expression and purification of F_1F_O ATP synthase

The *E. coli* F_1F_O ATP synthase was expressed using plasmid pBWU13β-His in *E. coli* DK8 cells as described[2]. The cultures were grown in a LEX-48 bioreactor (Epiphyte Three, Toronto, Canada) overnight at +37 °C. The cells were collected, washed with 10 mM Tris-HCl pH 8 and finally resuspended in 50 mM MOPS-NaOH pH 8, 100 mM NaCl, 5 mM $MgCl_2$, 30 g/l sucrose, 10 % glycerol and stored at -80°C.

Cells were disrupted by a high-pressure homogenizer HPL6 (MAXIMATOR AG, Düdingen, Switzerland) in the presence of DNase I, protease inhibitors PMSF, Pefabloc, and lysozyme. Undisrupted cell debris

was separated by centrifugation (12’000 g, 20 minutes, +4 °C). Membranes were collected by ultracentrifugation (235’000 g, 90 minutes, +4 °C) and resuspended in 50 mM MOPS-NaOH pH 8, 100 mM NaCl, 5 mM $MgCl_2$, 30 g/l sucrose, 10 % glycerol.

Membrane solubilization was done with 2 % Lauryl Maltose Neopentyl Glycol (LMNG, Anatrace, Maumee, US) for one hour at +4 °C. Not solubilized material separated with 200'000 x g, 30 min, +4 °C. Supernatant was collected, 5 mM Imidazole was added before loading on a 5 mL HisTrap FF column (Cytiva, Marlborough, US), pre-equilibrated with 50 mM MOPS-NaOH pH 8, 100 mM NaCl, 5 mM $MgCl_2$, 30 g/l sucrose, 10 % glycerol, 20 mM imidazole, 0.005 % LMNG. The column was washed twice with 5 CV of the same buffer containing 20 and 75 mM imidazole respectively, and the enzyme was eluted with the same buffer containing 285 mM imidazole. The peak fractions were pooled, concentrated, flash frozen in LN_2 and kept at –80 °C.

Expression and purification of GFP-ELP-SpyCatcher

GFP-ELP-SpyCatcher was expressed from plasmid pQE80L-SpyCatcher-ELP-GFP[3] (Addgene #69835) in *E. coli* C43 and grown in LB to an OD_{600} of 0.55 using a MaxQ™ 500 shaker (Thermo Fisher Scientific, Waltham, US). The ELP (elastin-like protein) was introduced by Bedbrock *et al*. to minimize steric interference between GFP and the SpyCatcher sequence[3]. After protein expression was induced with 2 mM IPTG (Santa Cruz Biotechnology, Dallas, US), the temperature was reduced to +27 °C and the cultures were incubated overnight at 220 rpm. Cells were harvested by centrifugation, washed with TBS, flash frozen in LN_2 and stored at –80 °C.

Cells were disrupted by a high-pressure homogenizer in the presence of DNase I, protease inhibitors PMSF, Pefabloc, and lysozyme. Undisrupted cell debris was separated by centrifugation (12’000 g, 20 minutes, +4 °C). The supernatant was loaded on Ni-NTA agarose beads (PureCube, Monheim, Germany) preequilibrated with TBS. The column was washed with TBS containing 20 mM imidazole (10 CV) and 40 mM imidazole (20 CV). Bound protein was eluted with TBS containing 250 mM

imidazole, concentrated (Amicon Ultra, 10 kDa cut-off, Merck, Darmstadt, Germany), and dialyzed three times against 2 L TBS over 36 hours.

Covalent coupling of SpyTag carrying proteorhodopsin variants and MBP-SpyCatcher variants

pR-SpyTag variants were resuspended at 20-50 µM concentration in 20 mM MOPS-BTP pH 7.25, 150 mM NaCl, 10 % glycerol, 1 % OG, 0.5 mM TCEP and mixed with a 1.2x molar excess of MBP-SpyCatcher or 2xMBP-SpyCatcher in PBS. OG from a 20 % stock solution was added until 1 % OG final concentration to compensate for the volume added of MBP-SpyCatcher variants. The reaction mixture was incubated for 2 hours at +25 °C and 1200 rpm. Coupled product was separated from uncoupled material by gel filtration (Superdex 200 Increase 10/300 GL, Cytiva, Marlborough, US) with 20 mM MOPS-BTP pH 7.25, 150 mM NaCl, 10 % glycerol, 1% OG, 0.5 mM TCEP.

Synthesis of trisNTA-SpyTag

Synthesis of tris-NTA

Synthesis of tris-NTA-*t*-Bu-NH_2 **2** was done according to previously reported procedures. See in particular: Z. Huang, P. Hwang, D. S. Watson, L. Cao, F. C. Szoka Jr., *Bioconjug Chem* **2009**, *20*, 1667–72. And references therein.

Peptide Synthesis

Peptides were synthesized manually using the following general procedure. The synthesis was performed using a TentaGel S Ram resin (loading: 0.25 mmol/g) in a 10 mL polypropylene syringe fitted with a polypropylene frit, a Teflon stopcock and a stopper. Before the start of the synthesis, the resin was swollen in DCM (5 mL, 10 min). After removal of DCM, the Fmoc-protecting group of the resin was removed by using a solution of 20 % piperidine in NMP followed by washing with NMP (3 x

4 mL), MeOH (3 x 4 mL) and DCM (3 x 4 mL). Coupling of amino acids was performed by using Fmoc-protected amino acids (3 eq.), Oxyma (3 eq.) and DIC (3 eq.) in NMP (5 mL) for 1 h before washing with NMP, MeOH and DCM (3 x 4 mL each). Stirring of the reaction mixture at any given step was performed by attaching the closed syringes to a rotating axis. The completion of the reaction was checked by using the TNBS test, and repeated if the resin was orange or red. Removal of the Fmoc-protecting group was performed by using a solution of 20 % piperidine in NMP (5 mL, 2 x 10 min). After filtration, the resin was washed with NMP (3 x 4 mL), MeOH (3 x 4 mL) and DCM (3 x 4 mL). Coupling of amino acids was performed by using Fmoc-protected amino acids (3 eq.), Oxyma (3 eq.) and DIC (3 eq.) in NMP (5 mL). The resin was stirred for 1 h before it was washed with NMP, MeOH and DCM (3 x 4 mL each).

TFA cleavage from resin and global deprotection were performed by adding a solution of TFA/TIS/DODT/H_2O (95:2:2:1, v/v/v/v, 20 mL/g resin) to the resin for 5 h. The peptide was precipitated with *t*-BuOMe, dissolved in H_2O/MeCN with 0.1 % TFA and subsequently purified by preparative RP-HPLC.

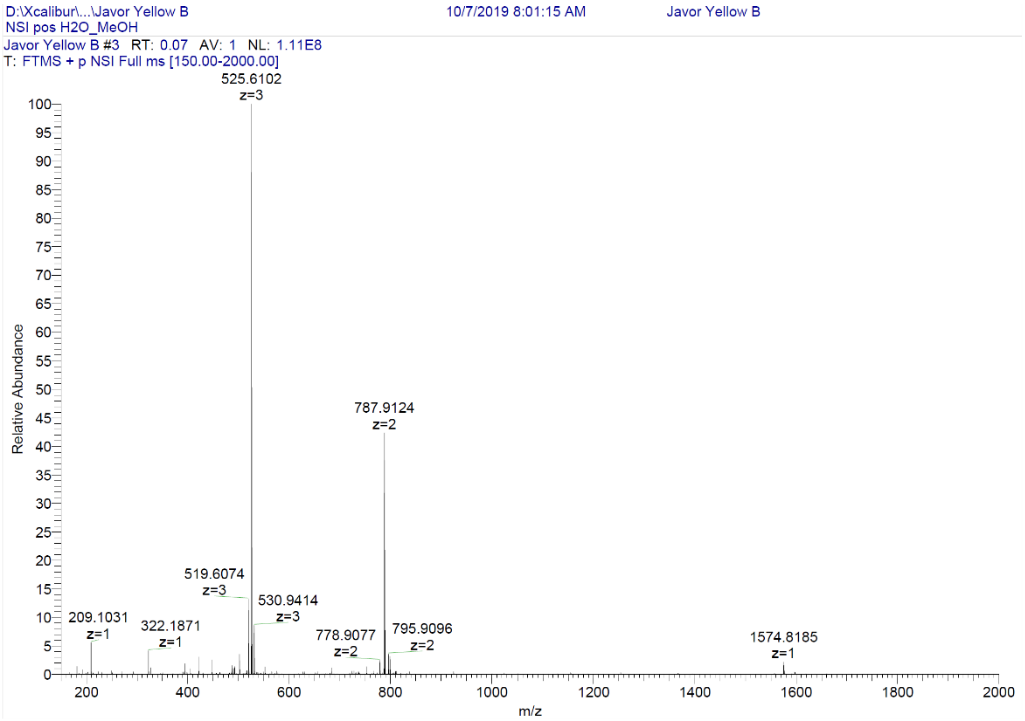

Synthesis of tris-NTA-t-Bu-ClAc 3

Tris-NTA-*t*-Bu-NH_2 **2** (0.10 g, 0.069 mmol) in dry CH_3CN (0.69 mL) was added to chloracetic anhydride (ClAc) (18 mg, 0.10 mmol, 1.5 eq.) and diisopropylethylamine (DIPEA) (36 µl, 27 mg, 0.21 mmol, 3 eq.) in dry CH_3CN (0.69 mL) at 0 °C and stirred at RT for 2 h. The solvents were evaporated, and the residue purified by silica gel flash chromatography (DCM:MeOH, 0 to 5 % MeOH) to yield tris-NTA-*t*-Bu-ClAc as a white sticky solid (57.9 mg, 55.1 %).

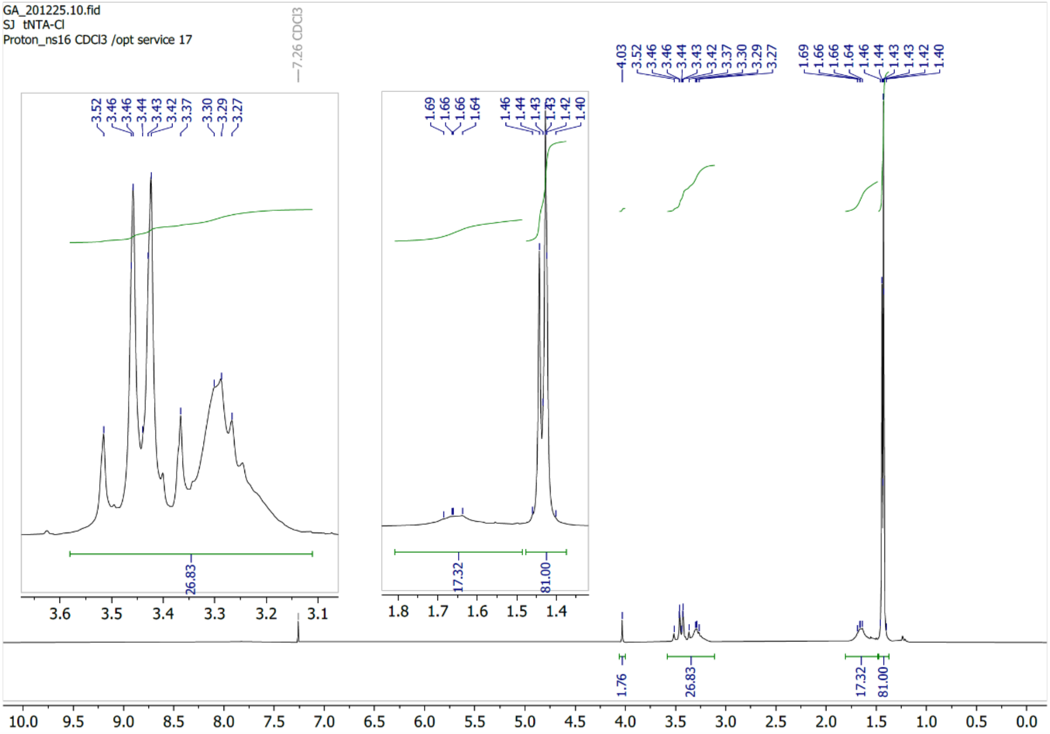

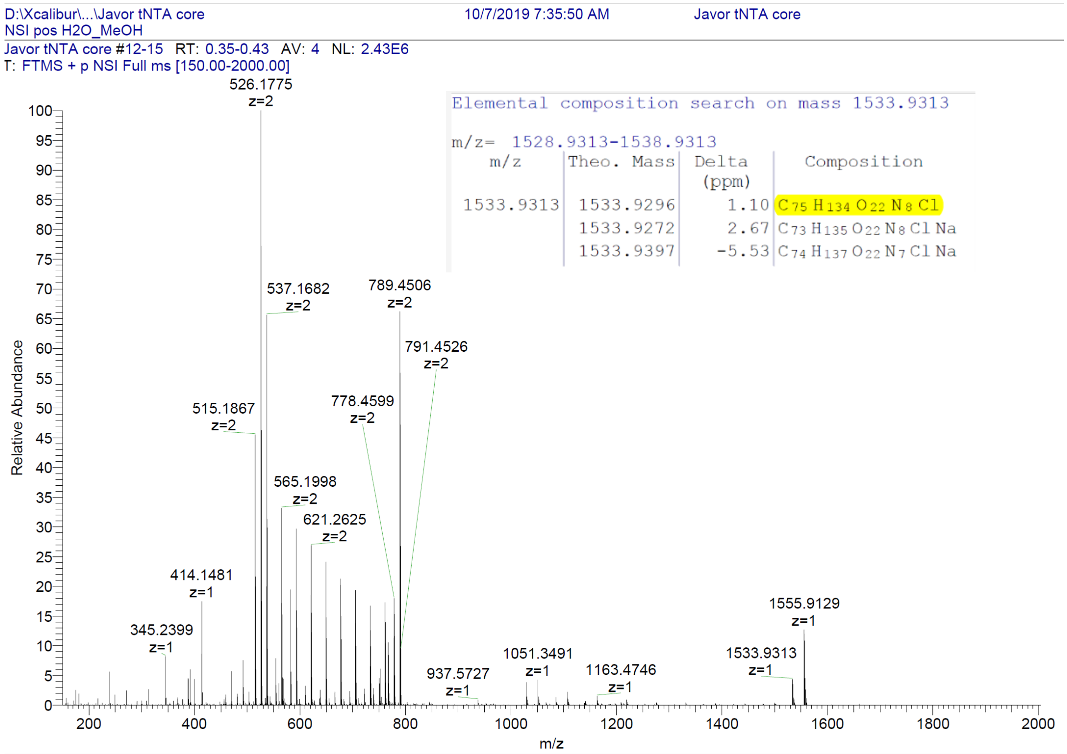

Synthesis of tris-NTA-ClAc 4

The *tert*-butyl ester **3** was stirred in CH_3Cl/TFA (2:1) (6.0 mL) for 5 h. The solvents were evaporated, and the tris-NTA-ClAc was purified by RP-HPLC and obtained in the form of TFA salt as a white solid (19.0 mg, 33.9 %) after lyophilization.

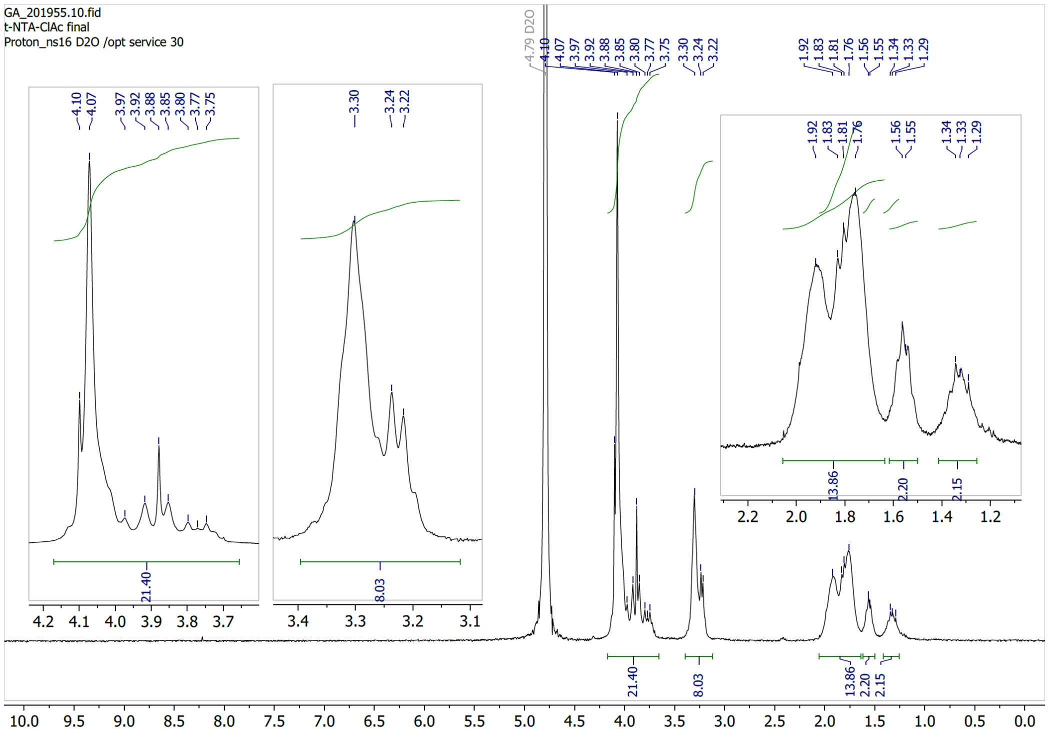

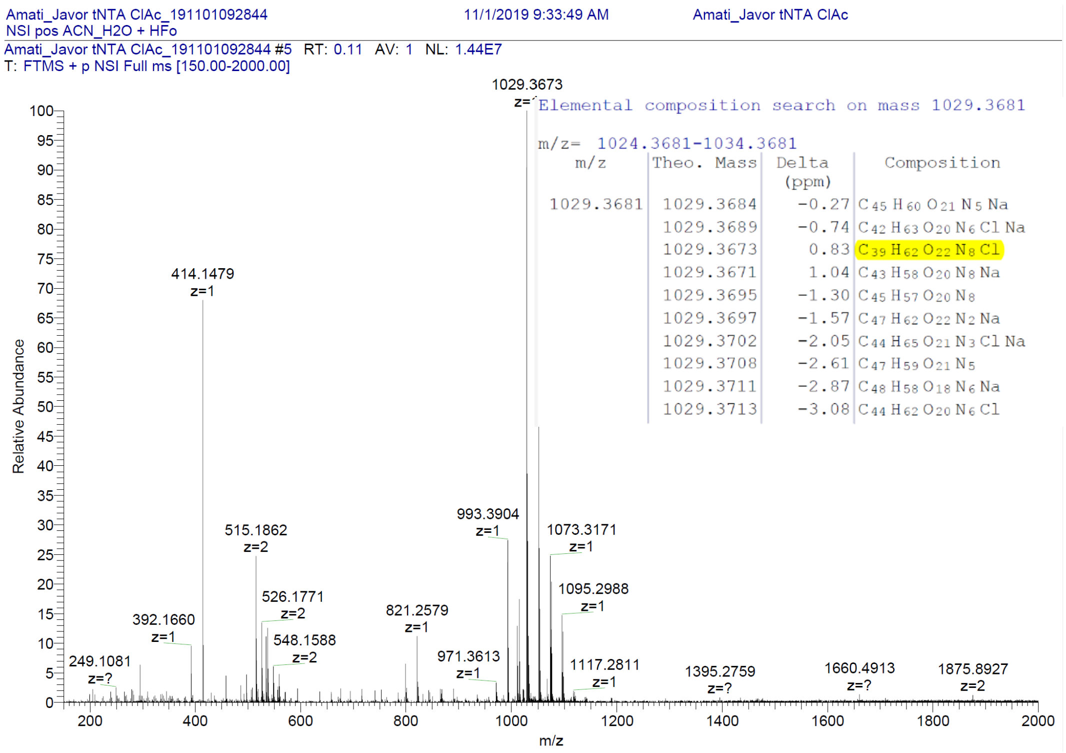

Synthesis of tris-NTA-SpyTag 5

Peptide SpyTag-Cys (AHIVMVDAYKPTKC-NH_2, 4.8 mg, 2.4 µmol) (5.0 mg/mL) in ammonium bicarbonate buffer (50 mM, pH 8.0), TCEP (1.0 mM) and tris-NTA-ClAc (7.4 mg, 5.0 µmol, 2.1 eq.) in CH_3CN/H_2O (3:1) (1.6 mL) were stirred for 16 h and the solvents were evaporated. Tris-NTA-SpyTag was obtained in the form of TFA salt as a fluffy white solid after RP-HPLC purification and lyophilization (3.2 mg, 0.92 µmol, 38 %).

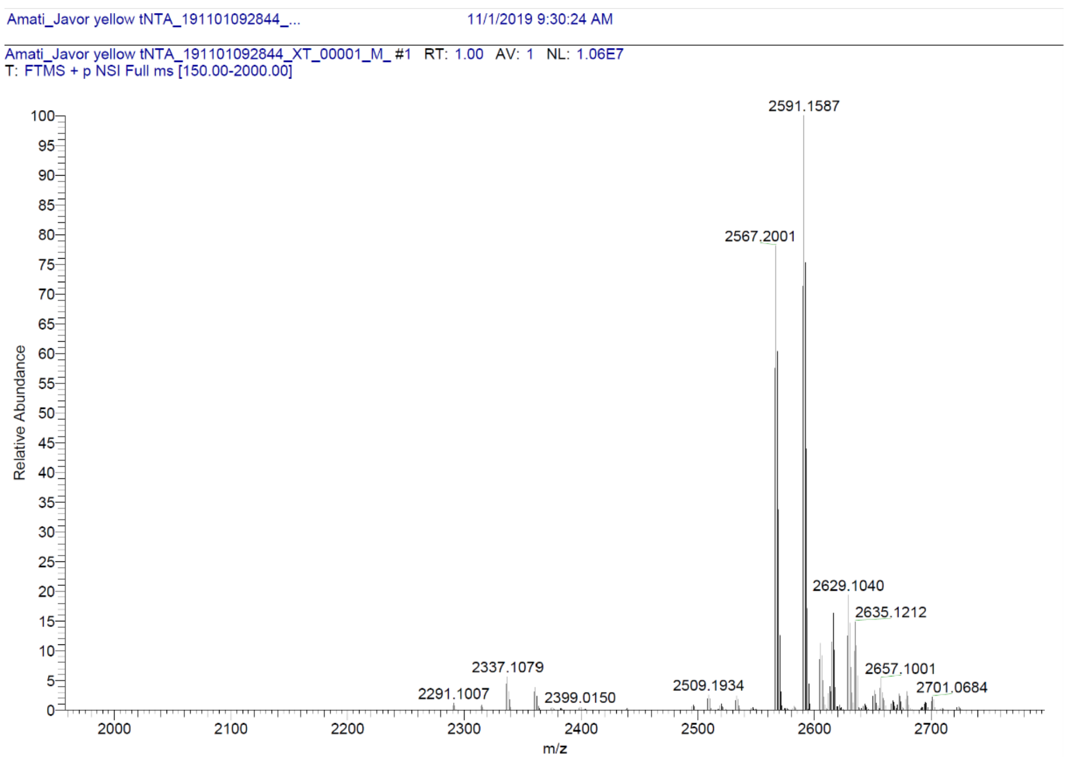

Non-covalent coupling of HisTagged proteins with bifunctional *tris*NTA-SpyTag linker

Coupling of *tris*NTA-SpyTag with SpyCatcher containing LSU

A portion of MBP-SpyCatcher or GFP-ELP-SpyCatcher (20 to 50 µM) was mixed with a 3-fold excess of compound 5 (*tris*NTA-SpyTag, dissolved in 20 % EtOH, 100 mM NaOH) and incubated for 2 h at +25 °C and 1200 rpm. Excess compound 5 was removed by a prepacked gel filtration column (CentriPure P10, emp Biotech, Berlin, Germany).

Labelling of *tris*NTA-MBP with DY647P1 NHS-ester

A portion of *tris*NTA-MBP (~ 20-50 µM) was mixed with a 10-times molar excess of DY647P1 NHS-ester (Dyomics, Jena, Germany) and incubated at +25 °C. After 30 minutes, 30 mM Tris-HCl pH 8 was added to quench the reaction, and excess DY647P1 was removed using a prepacked gel filtration column (CentriPure P10, emp Biotech, Berlin, Germany).

Reversible coupling of *tris*NTA-protein and proteins containing a HisTag

Non-covalent coupling of HisTagged protein with a *tris*NTA-protein complex was done in presence of 0.5 mM $NiSO_4$ and with a 5-times molar excess of *tris*NTA-MBP or *tris*NTA-GFP, if not otherwise indicated. The mixture was incubated for 15 minutes at +25 °C prior to reconstitution into liposomes.

Reconstitution of membrane proteins into preformed liposomes

Soybean lecithin lipids (Alfa Aesar, Ward Hill, US) were vigorously resuspended under nitrogen atmosphere in the according buffer at 10 mg/mL and subjected to seven freeze/thaw cycles (LN_2 / +29.4 °C). The resulting liposomes were made uniform in size by extrusion (21x) through a 100 nm Whatman filter (Cytiva, Marlborough, US). Liposomes were destabilized by 0.7 % OG (Glycon, Luckenwalde, Germany), pR was added (~ 20 enzymes per liposome) and the suspension was incubated for 30 minutes at room temperature with occasional gentle flicking. For proton pumping experiments, 2 mM 8-Hydroxypyrene-1,3,6-trisulfonic acid (pyranine; Thermo Fisher Scientific, Waltham, US) was included in the buffer. In co-reconstitution experiments, purified ATP synthase from *E. coli* (1-2 enzymes per vesicle) was added and the 30 min incubation was done on ice instead. OG was removed by prepacked gel filtration column (CentriPure PF10, emp Biotech, Berlin, Germany) and the obtained proteoliposomes were collected by centrifugation (200’000 g, 1 h, +4 °C) and resuspended at 10 mg/mL lipid.

Proton Uptake Measurements with ACMA

Inwards proton pumping by the ATP synthase was monitored by 9-amino-6-chloro-2-methoxyacridine (ACMA, Thermo Fisher Scientific, Waltham, US) as described[4] with some minor changes. Briefly, 20 to 50 µL proteoliposomes containing ATP synthase were added to 1.5 mL of HMK buffer (10 mM HEPES, pH 7.4, 2 mM $MgCl_2$, 100 mM KCl, 10 mM KNO_3), containing 1 µM ACMA and 100 nM valinomycin. After obtaining a stable baseline, proton pumping was initiated by addition of 1 mM Na_2ATP. After the

reaction had reached an equilibrium, the proton gradient was dissipated by addition of 60 mM NH_4Cl. Changes in ACMA fluorescence were monitored using 410 nm and 480 nm as excitation and emission wavelengths, respectively with a Cary Eclipse Fluorescence Spectrometer (Agilent Technologies, Santa Clara, US).

Light-driven proton pumping measurements

Twenty to fifty microliters proteoliposomes containing pR and encapsulated pyranine were added to 1.5 mL 1 mM MOPS-BTP pH 6.75, 50 mM KCl, 500 nM valinomycin and incubated for 20 minutes at room temperature. The pyranine signal (ratio of emission at 510 nm upon excitation at 406 and 460 nm) was measured using a Cary Eclipse Fluorescence Spectrometer (Agilent Technologies, Santa Clara, US) as described[2]. Proton pumping was initiated by illumination with a custom-fitted LED lamp (520 ± 20 nm, OSRAM LED ENGIN LuxiGen®, LZ4-40G108). The pH was calculated as described[2].

Membrane potential measurements

20-50 µL proteoliposomes were added to 1.5 mL 100 mM MOPS-BTP pH 6.75, 50 mM KCl, 20 mM NH_4Cl, 500 nM, 1,5-Bis-(5-oxo-3-propylisoxazol-4-yl)-pentamethin-oxonol (oxonol VI; AnaSpec, Fremont, US)[5]. The oxonol VI signal (excitation at 580 nm; emission at 660 nm) was measured with the Cary Eclipse Fluorescence Spectrophotometer (Agilent Technologies, Santa Clara, US). Proton pumping was initiated with the green LED as described above. After the time indicated, the membrane potential was dissipated by the addition of 500 nM valinomycin.

Light driven ATP production

20-50 µL proteoliposomes containing pR and ATP synthase were added to 500 µL 20 mM tris-phosphate pH 7.4, 5 mM $MgCl_2$, 0.2 mM ADP and 0.5 mg/mL luciferase/luciferin solution (ATP Bioluminescence Kit CLS II; Roche, Basel, Switzerland). Proton pumping activity of pR was initiated by

illumination with green light LED described. At indicated time points, illumination was stopped, and the luminescence of the reaction was measured with GloMax® 20/20 Luminometer (Promega, Madison, US). The measurements were quantified by addition of a standardized amount of ATP prior to the measurement as described[4].

Results and Discussion

Surface charge of proteorhodopsin influences the orientation into liposomes

The surface charge representation of proteorhodopsin (pdb: 2L6X) in Fig. S1 shows that the protein is more positively charged on side that is located on the inside of the cell (Negative side of the membrane) and is more negatively charged on the side that is directed towards the outside of the cell (positive side of membrane). This agrees with the general positive-inside rule found in membrane proteins. It has been found that reconstitution of pR into liposomes is influenced by electrostatic interactions, leading to a mainly right-side out orientation in liposomes containing negatively charged DOPG lipids and an inside out orientation in liposomes containing positively charged DOTAP lipids[18].

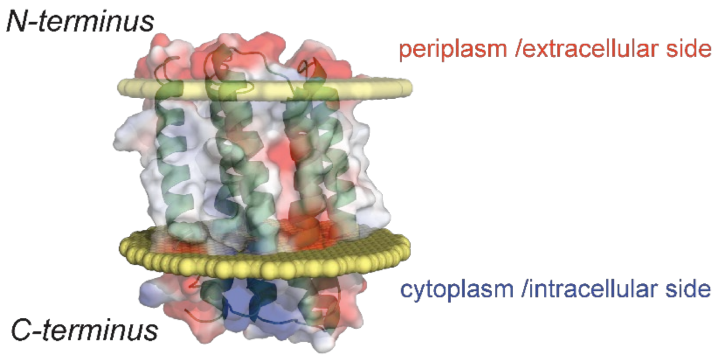

Figure S1. Surface charge representation of proteorhodopsin (pdb: 2L6X) showing the uneven distribution of charged residues on the cytoplasmic side (more positively charged, blue) and periplasmic (more negatively charged, red) side of the membrane. In unguided reconstitution, the

positively charged cytoplasmic surface preferably interacts with the membrane, leading to a right-side out orientation. Figure created with Pymol.

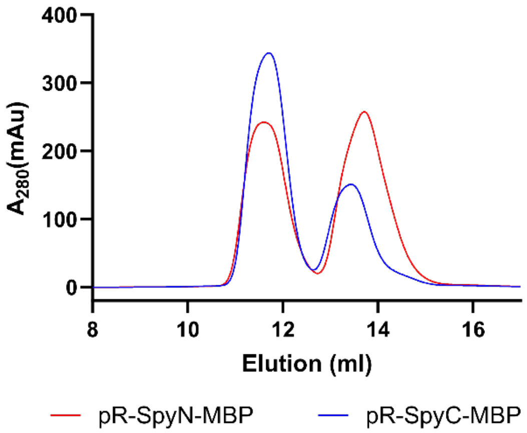

Figure S2. Profile of gel filtration chromatography after coupling reaction of pR-SpyN or pR-SpyC with MBP-SpyCatcher. The size of the products was verified using SDS PAGE (Figure 1C).

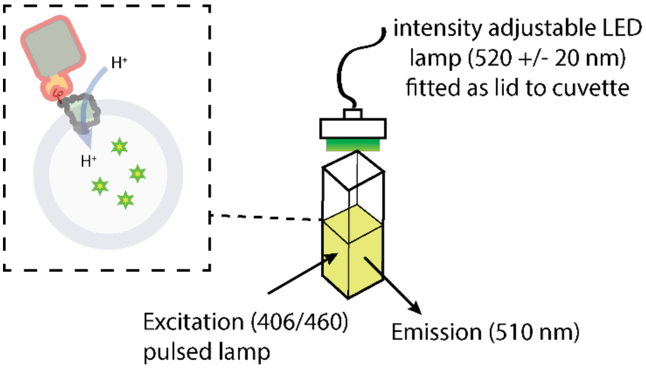

Figure S3. Setup for the pH change measurements. pR-MBP constructs were reconstituted into liposomes containing pyranine as pH sensitive dye and, diluted in buffer and placed into a cuvette in a flashlamp-based fluorescence spectrophotometer using alternatingly 406 and 460 nm as excitation wavelengths and 510 nm as emission wavelength. The sample was irradiated from top with a custom-built LED lamp (OSRAM LED ENGIN LuxiGen®, LZ4-40G108).

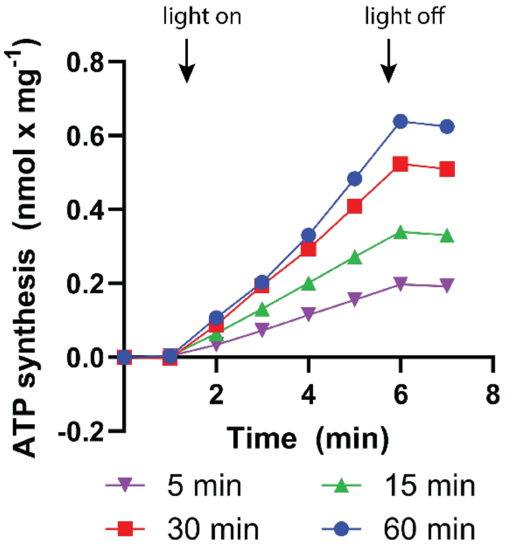

Figure S4. Dependence of the ATP synthesis rate on the time of incubation of the pR-SpyC-MBP and ATP synthase with preformed liposomes. After the indicated time, detergent was removed using a gravity gel filtration column with a 25 kDa cutoff. (n=1)

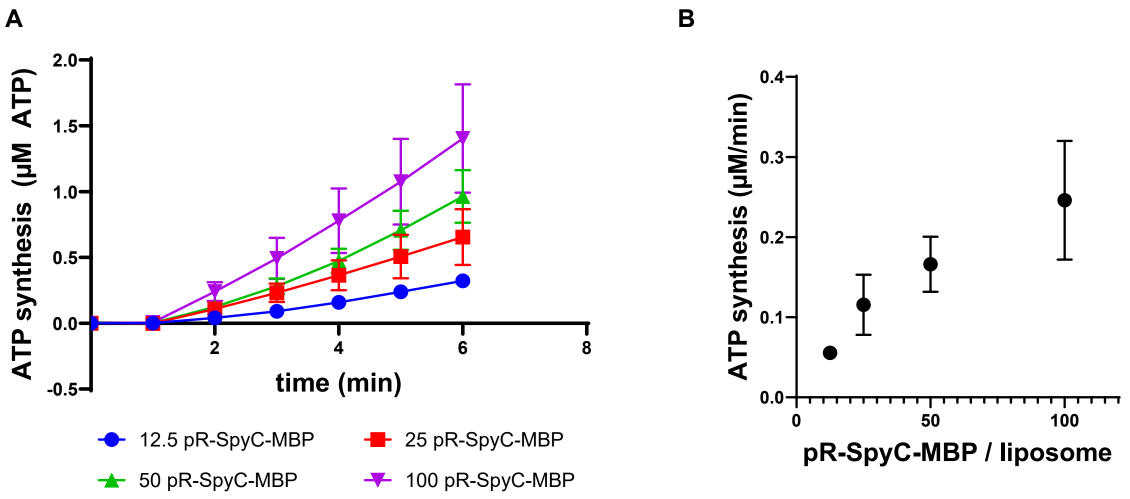

Figure S5. Variation of pR to ATP synthase ratio for light-driven ATP synthesis. (A) Different amounts of pR-SpyC-MBP were co-reconstituted with 3 ATP synthase on average per liposome. Light-driven ATP synthesis was initiated after 1 minute and measured using luciferin/luciferase assay. (n=3, n=2 for 12.5 pR) (B) ATP synthesis rates derived from Figure S10 A plotted as a function of pR-SpyC-MBP content per liposome

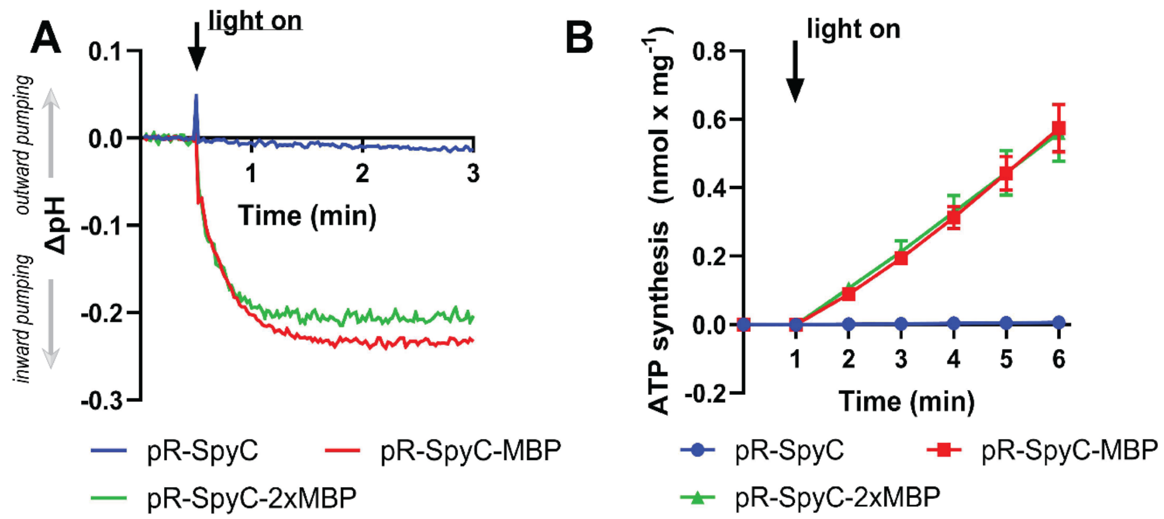

Figure S6. Effect of large soluble unit (LSU) size on proton pumping and ATP synthesis activity. (A) purified pR-SpyC alone or coupled to MBP-SpyCatcher or 2xMBP-SpyCatcher (containing two MBP units) was reconstituted into proteoliposomes containing pyranine and light-induced proton influx was measured. (B) as A, but ATP synthase was co-reconstituted and light-driven ATP synthesis was measured using luciferin/luciferase assay. (n=3)

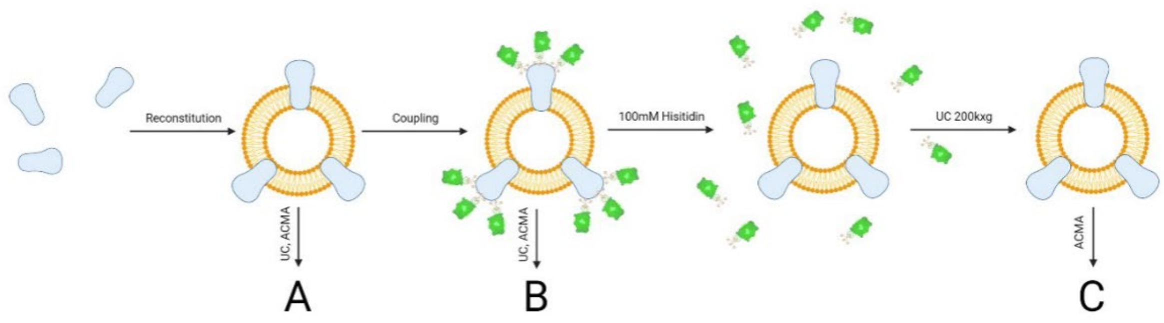

Figure S7. Overview of experiment confirming the binding of *tris*NTA-GFP to the HisTag of ATP synthase reconstituted into liposomes. ATP synthase was reconstituted into liposomes (sample A), mixed with *tris*NTA-GFP and subjected to ultracentrifugation to remove excess *tris*NTA-GFP (sample B). Subsequently, it was treated with 100 mM Histidine and subjected to ultracentrifugation to remove released *tris*NTA-GFP (sample C). Samples B and C were analyzed for GFP fluorescence and samples A, B, C were analyzed for protein activity (ACMA). Scheme created with biorender.com.

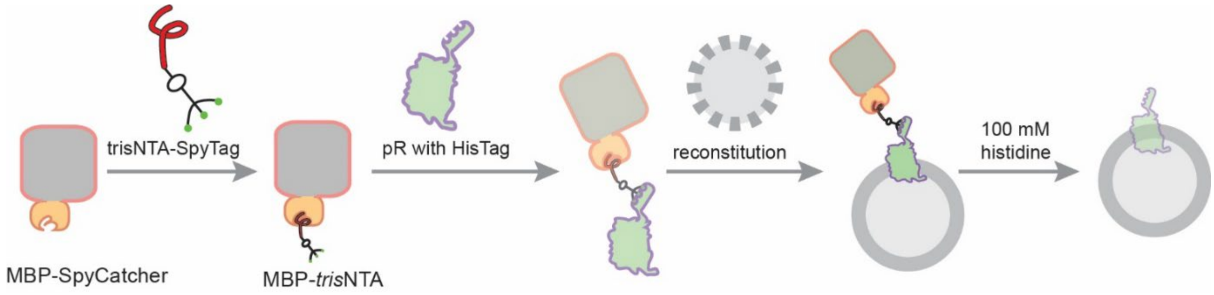

Figure S8. Strategy for orientation with reversibly bound *tris*NTA-MBP to proteorhodopsin carrying a C-terminal HisTag. MBP-SpyCatcher is functionalized with the bifunctional linker *tris*NTA-SpyTag forming the transient LSU MBP-*tris*NTA. This construct is mixed with proteorhodopsin carrying a C-terminal HisTag and used in a reconstitution experiment with preformed liposomes and detergent, where the latter is removed using a gel filtration column. Addition of 100 mM histidine removes the LSU and the proteoliposomes carrying native proteorhodopsin can be harvested by centrifugation.

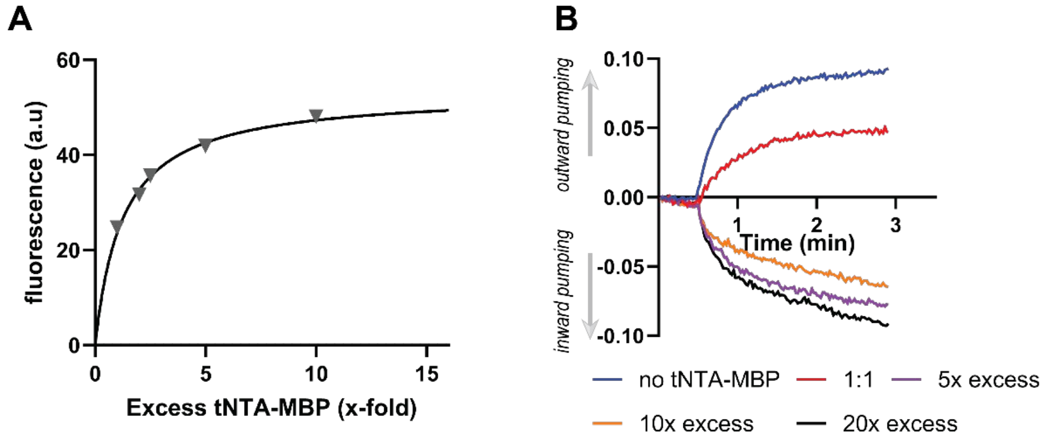

Figure S9. Variation of *tris*NTA-SpyTag over pR with a C-terminal His-Tag. Prior to reconstitution, the transient LSU was mixed in varying molar ratios with His-tagged pR whose concentration was kept constant. Detergent was removed using gel filtration and the liposomes were collected using ultracentrifugation. (A) Here, MBP-*tris*NTA was fluorescently labelled with DY-647and the fluorescence intensity of the pellet after ultracentrifugation was measured. (n=2) (B) Like A, but non-fluorescent MBP was used, and light-driven proton pumping was determined by monitoring pyranine fluorescence. (n=2)

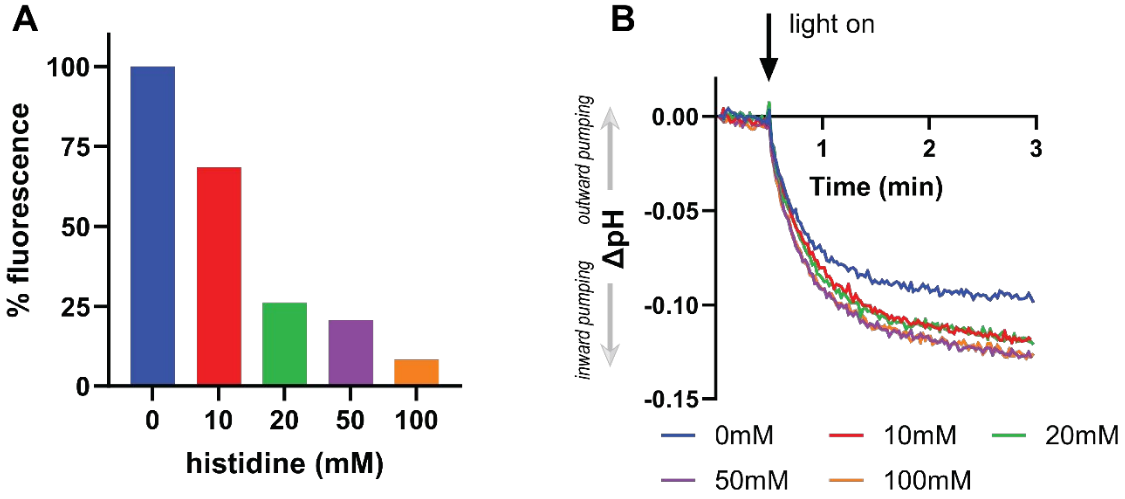

Figure S10. Removal of transiently bound LSU using histidine. (A) pR with a C-terminal HisTag was mixed with *tris*NTA-MBP labelled with DY-647 (ratio 1:5) and reconstituted into liposomes. The proteoliposomes were split into 5 portions and mixed with the indicated histidine concentrations for 15 min. Liposomes were collected using ultracentrifugation and the DY-647 fluorescence intensity in the pellet was determined. (n=2) (B) Same as A, but non-fluorescently labelled *tris*NTA was used, and the light-driven proton pumping activity was determined by monitoring pyranine fluorescence. (n=2)

References

(1) Veggiani, G.; Nakamura, T.; Brenner, M. D.; Gayet, R. V.; Yan, J.; Robinson, C. V.; Howarth, M. Programmable Polyproteams Built Using Twin Peptide Superglues. *Proc Natl Acad Sci U S A* **2016**, *113* (5), 1202–1207. https://doi.org/10.1073/pnas.1519214113.

(2) Wiedenmann, A.; Dimroth, P.; von Ballmoos, C. Δψ and ΔpH Are Equivalent Driving Forces for Proton Transport through Isolated F0 Complexes of ATP Synthases. *Biochimica et Biophysica Acta (BBA) - Bioenergetics* **2008**, *1777* (10), 1301–1310. https://doi.org/10.1016/j.bbabio.2008.06.008.

(3) Bedbrook, C. N.; Kato, M.; Ravindra Kumar, S.; Lakshmanan, A.; Nath, R. D.; Sun, F.; Sternberg, P. W.; Arnold, F. H.; Gradinaru, V. Genetically Encoded Spy Peptide Fusion System to Detect Plasma Membrane-Localized Proteins In Vivo. *Chemistry & Biology* **2015**, *22* (8), 1108–1121. https://doi.org/10.1016/j.chembiol.2015.06.020.

(4) von Ballmoos, C.; Biner, O.; Nilsson, T.; Brzezinski, P. Mimicking Respiratory Phosphorylation Using Purified Enzymes. *Biochim Biophys Acta* **2016**, *1857* (4), 321–331. https://doi.org/10.1016/j.bbabio.2015.12.007.

(5) Belevich, N.; Von Ballmoos, C.; Verkhovskaya, M. Activation of Proton Translocation by Respiratory Complex I. *Biochemistry* **2017**, *56* (42), 5691–5697. https://doi.org/10.1021/acs.biochem.7b00727.
